# GAGrank: Software for Glycosaminoglycan Sequence Ranking Using a Bipartite Graph Model

**DOI:** 10.1016/j.mcpro.2021.100093

**Published:** 2021-05-14

**Authors:** John D. Hogan, Jiandong Wu, Joshua A. Klein, Cheng Lin, Luis Carvalho, Joseph Zaia

**Affiliations:** 1 Program in Bioinformatics, Boston University, Boston, Massachusetts, USA; 2 Department of Biochemistry, Center for Biomedical Mass Spectrometry, Boston University School of Medicine, Boston, Massachusetts, USA; 3 Department of Mathematics & Statistics, Boston University, Boston, Massachusetts, USA

**Keywords:** glycosaminoglycan, tandem mass spectrometry, bipartite graph, sequencing, CS, chondroitin sulfate, DS, dermatan sulfate, EDD, electron detachment dissociation, ExD, electron activated dissociation, GAG, glycosaminoglycan, HOST, heparin oligosaccharide sequencing tool, HS, heparan sulfate, KS, keratan sulfate, MS^2^, tandem mass spectrometry, NETD, negative electron transfer dissociation, SA, simulated annealing

## Abstract

The sulfated glycosaminoglycans (GAGs) are long, linear polysaccharide chains that are typically found as the glycan portion of proteoglycans. These GAGs are characterized by repeating disaccharide units with variable sulfation and acetylation patterns along the chain. GAG length and modification patterns have profound impacts on growth factor signaling mechanisms central to numerous physiological processes. Electron activated dissociation tandem mass spectrometry is a very effective technique for assigning the structures of GAG saccharides; however, manual interpretation of the resulting complex tandem mass spectra is a difficult and time-consuming process that drives the development of computational methods for accurate and efficient sequencing. We have recently published GAGfinder, the first peak picking and elemental composition assignment algorithm specifically designed for GAG tandem mass spectra. Here, we present GAGrank, a novel network-based method for determining GAG structure using information extracted from tandem mass spectra using GAGfinder. GAGrank is based on Google’s PageRank algorithm for ranking websites for search engine output. In particular, it is an implementation of BiRank, an extension of PageRank for bipartite networks. In our implementation, the two partitions comprise every possible sequence for a given GAG composition and the tandem MS fragments found using GAGfinder. Sequences are given a higher ranking if they link to many important fragments. Using the simulated annealing probabilistic optimization technique, we optimized GAGrank’s parameters on ten training sequences. We then validated GAGrank’s performance on three validation sequences. We also demonstrated GAGrank’s ability to sequence isomeric mixtures using two mixtures at five different ratios.

## Highlights

• GAGfinder assigns glycosaminoglycan EDD and NETD product ions.
• GAGrank assigns the most probable sequence from the tandem MS.
• GAGrank ranks nodes using a bipartite network’s structure.
• GAGrank ranks glycosaminoglycan sequences based on their importance in the network.

## Graphical abstract

**Figure:**
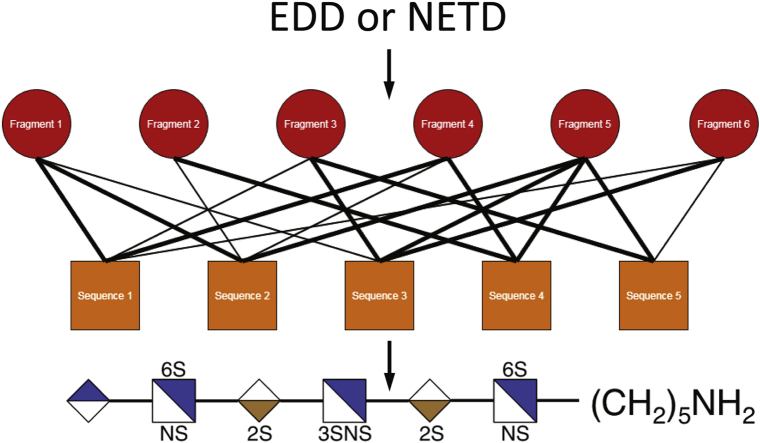

## In Brief

We demonstrate GAGrank, an algorithm that uses a bipartite graph model for sequencing glycosaminoglycans from EDD or NETD tandem mass spectra. The process involves first assigning glycosaminoglycan product ions using the GAGfinder algorithm. The second step is to rank possible sequences using GAGrank. We show GAGrank’s ability to sequence isomeric mixtures.

**Fig. 1:**
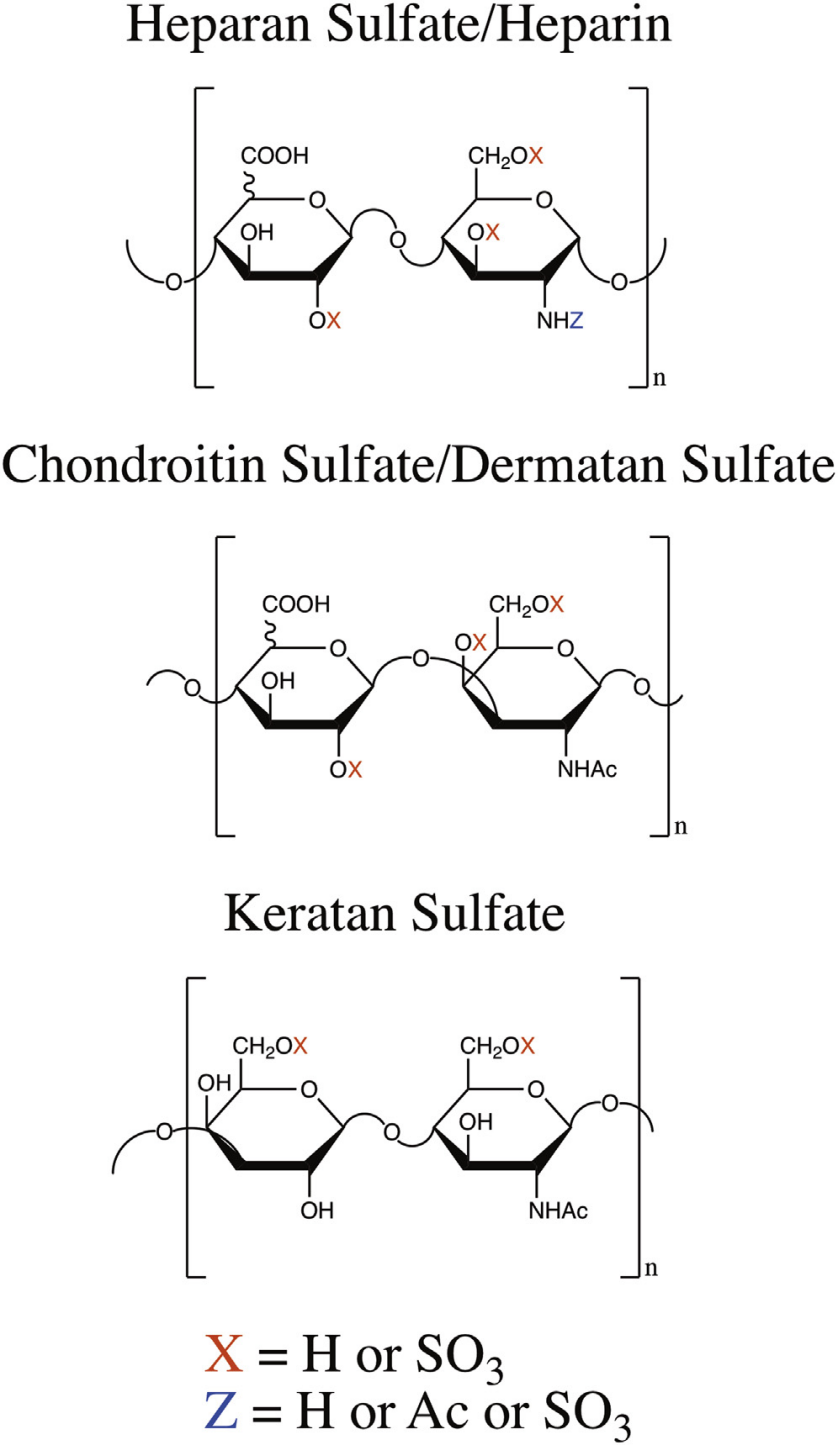
**Repeating disaccharide unit for each GAG class.** Each glycosaminoglycan class has its own unique repeating disaccharide unit that has distinct linkages and modification positions. Heparan sulfate (HS) can have up to four sulfates per disaccharide, chondroitin sulfate/dermatan sulfate can have up to three sulfates per disaccharide, and keratan sulfate can have up to two sulfates per disaccharide. HS and chondroitin sulfate/dermatan sulfate can have one of two C5 epimers as their uronic acid: glucuronic or iduronic acid. HS is the only class that can have hexosamine residues that are not *N*-acetylated.

The sulfated glycosaminoglycans (GAGs) are long, linear polysaccharides that can be found as the glycan portion of proteoglycans on cell surfaces and in extracellular matrices. There are three classes of sulfated GAGs, each with its own distinct repeating disaccharide unit (Fig. 1) and biology. Heparan sulfate (HS) participates in or affects blood coagulation (1), growth factor signaling (2), angiogenesis (3), and cell proliferation and migration (4). Chondroitin sulfate (CS), and the closely related dermatan sulfate (DS), participates in or affects brain development (5), spinal cord injury and neuroregeneration (6), neural stem cell migration (7), and osteoarthritis (8). Keratan sulfate (KS) participates in or affects corneal hydration (9), infection and wound repair (10), and cell migration (11). As a part of membrane proteoglycans and the extracellular matrix, GAGs bind numerous growth factors and growth factor receptors and thereby mediate cell–cell, cell–matrix, and host–pathogen interactions. As such, the ability to sequence GAGs quickly and accurately is an important step in understanding how changes in GAG sequences alter biological mechanisms.

We and others have demonstrated the effectiveness of electron activated dissociation tandem MS for sequencing GAG saccharides (12, 13, 14, 15, 16, 17, 18, 19, 20, 21, 22). Interpretation of the tandem mass spectra requires first assignment of product ion charge states and monosaccharide compositions. This requires a solution that can handle the varying elemental compositions of the product ions that make application of a simple averagine decomposition model impractical. Next, the most likely GAG sequence(s) must be assigned to the product ion pattern. In a typical MS^2^ experiment, spectra are preprocessed depending on the type of mass analyzer used. For ion cyclotron resonance and Orbitrap analyzers, the signal is first transformed from the time domain to the frequency domain, then calibrated to produce *m/z* domain spectra. The conversion of the *m/z* values to neutral masses requires special consideration for the GAG classes. Algorithms based on THRASH (23) for identification of monoisotopic peaks and estimation of elemental compositions do not suffice for GAGs because the sulfur and oxygen contents vary significantly among fragment ions, thus precluding use of a single averagine for elemental composition approximation. Our group recently developed a GAG-specific algorithm performing both of these steps, GAGfinder (18). GAGfinder provides a list of peaks and annotations from an MS^2^ experiment for the sequencing pipeline.

Electron activated dissociation (ExD) is a general term that refers to use of ion–ion or ion–electron reactions to dissociate the analyte. For fragmentation of anionic species, including GAGs, ExD includes electron detachment dissociation (EDD) (24), where the analyte is fragmented by detaching an electron from the analyte with a high-energy electron beam, and negative electron transfer dissociation (NETD) (25), where the analyte is fragmented by transferring an electron from the anionic precursor to a cationic radical reagent. Wolff and colleagues first demonstrated the efficacy of ExD for dissociating GAG oligosaccharides in various applications, using both EDD (14, 15, 26) and NETD (13). Huang and colleagues have also shown the utility of ExD for GAG oligosaccharides in terms of reducing labile sulfate loss (20). Clearly, ExD methods show promise as the analytical tool of choice in GAG sequencing.

Existing methods for computational GAG sequencing trace their origins to the heparin oligosaccharide sequencing tool (HOST) (27), published in 2005. HOST was developed as a Microsoft Excel workbook designed as an interface that integrates disaccharide information with MS^2^ data for sequencing of heparin/HS enzymatic digests. An update to GlycoWorkbench by Tissot *et al.* (28) calculates elemental compositions for GAG sequences and facilitates interpretation of GAG mass spectra by calculating *m/z* values and annotating fragment ions (29). In 2010, Spencer and colleagues published a method for estimation of the domain structure of HS chains based on disaccharide analysis and user-defined biosynthesis rules (30). This method uses three modular *in silico* steps: HS chain generation, HS chain digestion, and HS chain sorting based on domain matching. In 2014, Hu and colleagues published HS-SEQ (19), the first *de novo* sequencing tool for HS oligosaccharides. Based on a user-submitted MS^2^ fragment ion list and HS backbone information, HS-SEQ outputs probabilities for modifications at each position along the chain using a spectrum graph model. In 2015, Chiu and colleagues published the first database search application for GAG sequencing, GAG-ID (31). GAG-ID automates the interpretation of permethylated HS LC/MS^2^ data using a multivariate hypergeometric distribution with detected peaks separated into high-, medium-, and low-intensity bins. The same group later used a multivariate mixture model to determine GAG-ID identification accuracy given database search scores and ambiguity values among identifications (32). Finally, Duan and colleagues recently published over two publications a method for interpreting CS GAG MS^2^ data that uses a genetic algorithm to assign a likelihood score to each sequence for a given MS^2^ spectrum (33, 34).

These computational methods have succeeded in making GAG sequencing faster and easier, but they each have drawbacks. The first three—HOST, the Tissot method, and the Spencer method—use the results of disaccharide analysis to guide their algorithm, meaning that the methods are inappropriate for top- or middle-down glycomics studies. HS-SEQ shows promise in locating site-specific sulfation for HS oligosaccharides but requires prior knowledge about the HS backbone and does not handle mixtures as would be seen in an LC-MS^2^ experiment. Furthermore, it only considers HS oligosaccharides and does not work on CS or KS GAGs. GAG-ID shows promise in ranking individual GAG sequences mapping to a given GAG composition but requires an extensive chemical workup involving permethylation, desulfation, and pertrideuteroacetylation. Duan and Amster’s genetic algorithm shows great promise for reducing search space and computation time, but it is a nondeterministic algorithm and therefore cannot guarantee to reach a global optimum. We sought to develop a novel, deterministic GAG sequencing method that has fewer steps before use than the existing methods but still delivers optimal performance.

**Fig. 2:**
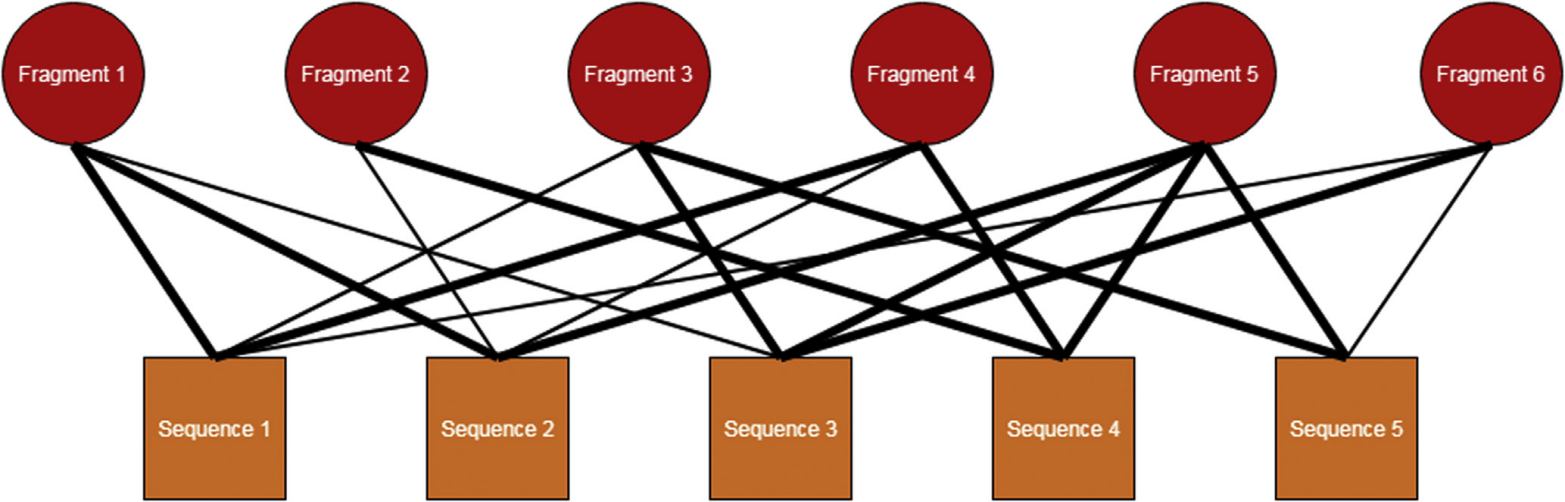
**Example bipartite network of sequences and fragments.** This is a toy visualization of a bipartite network of sequences and fragments. In this case, there are five sequences and six fragments. An edge between a sequence and a fragment denotes that fragment being a possible fragment for that sequence. The edge width denotes the type of fragment for that sequence; a wider edge represents a terminal fragment, whereas a narrower edge represents an internal fragment.

At the core of any sequencing method using MS^2^ data is the relationship between the unknown sequence and its fragments: the actual sequence is ascertained based on the fragment ions generated in the fragmentation process. For GAGs, there are often many possible sequences for a given composition, and in an ExD experiment, there is a rich complement of product ions in the spectrum. The relationship between possible sequences and observed product ions is many-to-many, and can be represented in a network structure. In particular, the network structure is that of a bipartite network, which is a network whose nodes can be separated into two distinct partitions with edges only connecting nodes in one partition to nodes with the other partition. Figure 2 shows a graphical representation of the bipartite network relationship between potential sequences and product ions.

The determination of node importance has been a topic of significant interest in network analysis, in particular for social networks (35), protein–protein interaction networks (36), and the World Wide Web (37), among many others. The concept of centrality in network analysis aims to solve this problem, and there are numerous existing algorithms for computing centrality measures. One such method is PageRank (38), developed by Brin and Page in 1996 for Google as a way to rank webpages according to their importance for search engine optimization purposes. Briefly, PageRank gives webpages higher importance values if they are linked to by other important webpages. PageRank was developed for general networks (*i.e.*, not bipartite networks), but a recent method, BiRank (39), was developed that adapts the PageRank algorithm for the specific case of bipartite networks. Briefly, BiRank gives nodes in partition A higher importance if they are linked to important nodes in partition B, and vice versa. Because of its design for bipartite networks, we employed BiRank with the goal of determining precursor sequence based on fragmentation patterns in the first GAG sequencing method developed using a network structure and network analysis algorithm, GAGrank. GAGrank was developed as a command line interface in the Python language (v3.8.1), and its repository can be cloned *via* Github at http://www.bumc.bu.edu/msr/software/. This paper describes the method and demonstrates its performance on a set of GAG standards.

## Experimental Procedures

### GAGrank Overview

**Fig. 3:**
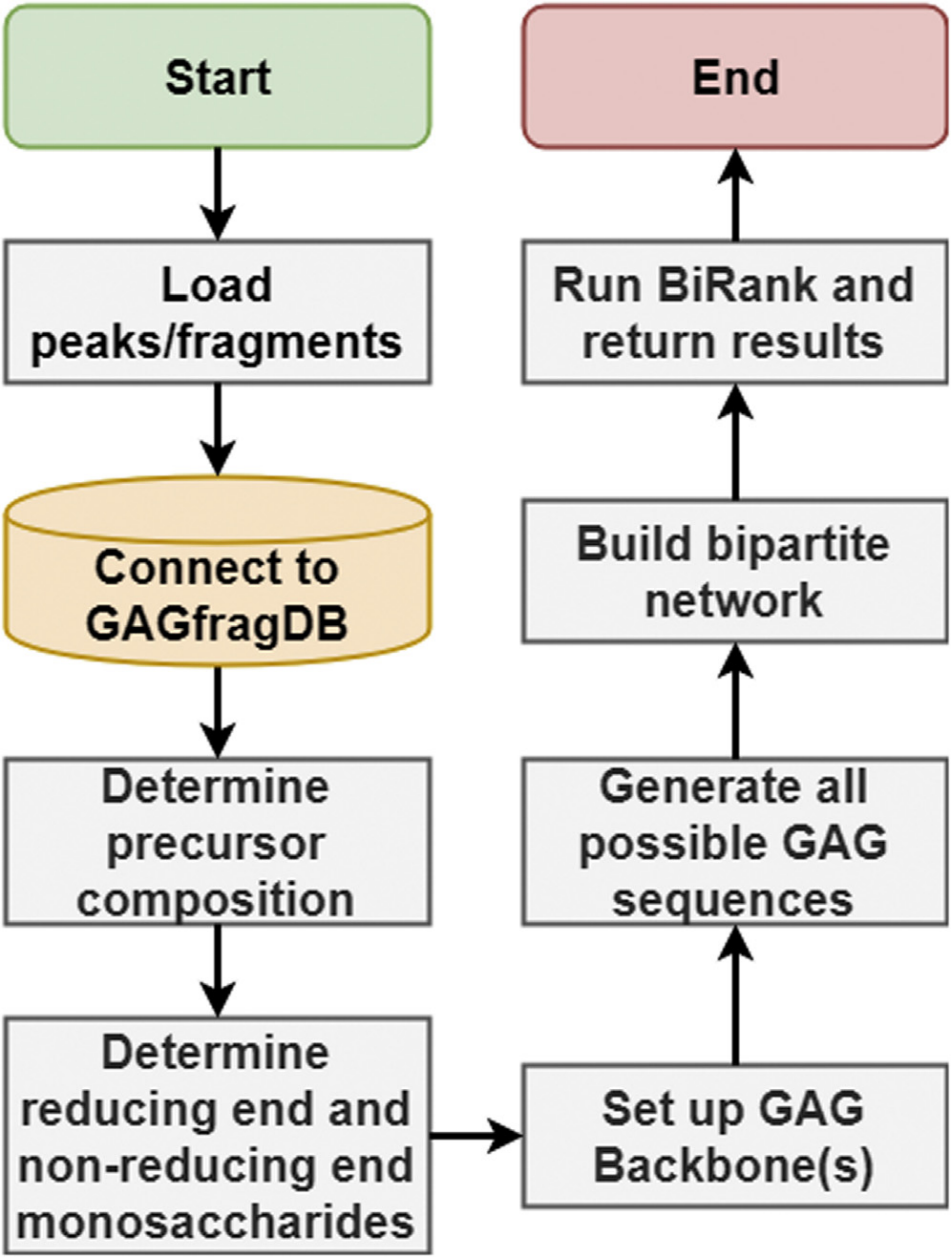
**Workflow for GAGrank algorithm.** The steps in GAGrank’s algorithm.

Figure 3 shows the steps in the GAGrank algorithm, the details of which are presented in the next several subsections.

#### Inputs

GAGrank has several inputs, both required and optional. The required inputs are the peak/fragment list, the GAG class, the precursor ion *m/z*, and the precursor ion charge, assuming unadducted deprotonated ions. The peak/fragment list must be an output data file from our previous work, GAGfinder (18), and the GAG class being analyzed must be denoted by its initials (*i.e.*, HS, CS, or KS). Since DS is a class of CS, the initials CS are required for these GAGs. The optional inputs are the reducing end tag and the number of sulfate losses to consider. If the analyte was tagged on the reducing end to break potential molecular symmetry, the user must denote what elements the tag adds to the sequence. For instance, if the reducing end tag is 4-nitrophenol, the tag should be encoded as C_6_H_3_NO_2_ rather than the 4-nitrophenol elemental composition of C_6_H_5_NO_3_, since that is the number of each element that is added to the structure. For deciding an appropriate number of sulfate losses for GAGrank to use, we recommend using the floor of two times the free proton index described in (40). However, this input is left to the user’s discretion.

#### Step 1: Load Peak/Fragment List

First, GAGrank loads the peak/fragment list returned by GAGfinder into Python as a NumPy array with two columns, fragment and G-score. The G-score is GAGfinder’s goodness-of-fit score for fitting experimental isotopic distributions found in spectra to theoretical isotopic distributions. A smaller G-score represents a better fit between the two distributions.

#### Step 2: Determine Precursor Composition

Next, GAGrank utilizes the database GAGfragDB to determine the precursor composition in a manner similar to GAGfinder. GAGfragDB was developed in SQLite to store every possible fragment for a given precursor composition, but it also stores useful information about precursors, such as their chemical formula and monoisotopic mass. GAGrank selects the precursor composition by comparing the neutral mass of the spectral precursor ion to the list of neutral masses in GAGfragDB and picking the one that is arithmetically closest. Further detail concerning GAGfragDB is present in our GAGfinder paper (18).

#### Step 3: Determine Reducing End and Nonreducing End Monosaccharides, if Possible

The next step in GAGrank’s pipeline is also similar to the one found in GAGfinder. By evaluating the number and type of monosaccharides present in the precursor’s composition, we can potentially determine the order of the monosaccharides in the oligosaccharide backbone. For a detailed description of this process, see (18).

#### Step 4: Set Up GAG Backbone(s)

We can build the backbone(s) of the GAG sequence using our understanding of GAG sequence construction and the terminal sugar residues determined in step 3. In the event that we cannot determine the terminal sugar residues, we must consider two backbones; one with amino sugars in the odd positions in the backbone and another with amino sugars in the even positions in the backbone. Given the backbone(s) of sugar residues and the GAG class for the structure, we can define the positions for potential modifications (Fig. 1).

#### Step 5: Generate All Possible GAG Sequences

We now have the backbone(s) of the GAG, the potential modification positions along the backbone(s), and the number of each modification (sulfation and acetylation). We use combinatorics to generate each possible sequence for a given composition.

#### Step 6: Build Bipartite Network

Using Python’s NetworkX module (41), we encode the relationships between each potential sequence and each fragment found by GAGfinder in a bipartite network. For each potential sequence, we derive its potential fragments by generating all terminal glycosidic fragments, terminal cross-ring fragments, and internal glycosidic–glycosidic fragments. We do not consider internal glycosidic–cross-ring or internal cross-ring–cross-ring fragments because they are rare and of low intensity, do not add much additional information about the sequence (and, in fact, may actually hurt the scoring because of coincidental matches), and increase computational time. We then compare this list of potential fragments to those found in the spectrum loaded in step 1 and place edges between the sequence and each fragment in the intersection. Equation 1 shows how we encode the edge width for these edges. The values for Equation 1 are based on those used in our previous work, HS-SEQ (19). In cases where a fragment could be both a terminal fragment and an internal glycosidic–glycosidic fragment, the edge width is selected as a terminal fragment. The tuning parameter *r1* controls the effect that double glycosidic bond fragments has on the performance of BiRank.

(1) $$w_{xy}=\left\{ \begin{array}{cc} 1.0 & \text{if}\;\text{fragment}\;x\;\text{is}\;\text{a}\;\text{terminal}\;\text{fragment}\;\text{in}\;\text{sequence}\;y\\ 0.2^{r1} & \text{if}\;\text{fragment}\;x\;\text{is}\;\text{an}\;\text{internal}\;\text{double}\;\text{glyosidic}\;\text{fragment}\;\text{in}\;\text{sequence}\;y \end{array}\right.$$

#### Step 7: Run BiRank and Return Results

The final step in GAGrank’s pipeline is to run the BiRank algorithm (39) on the network built in step 6. The pseudocode for BiRank is in Algorithm 1.

**Table:** Algorithm 1: BiRank Algorithm, adapted from (39)

**Table:** 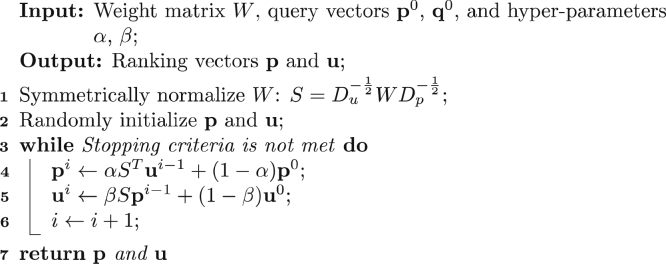

The inputs for BiRank include the graph’s weight matrix *W*, query vectors **p**^0^ and **u**^0^, and hyper-parameters α and β. The weight matrix is symmetric, consisting of the edge weights between nodes in the graph, as described in Equation 1. For pairs of nodes with no edge between them, the weight *w*_*ij*_ is given as 0. The query vectors store a prior belief about the ranking criterion for the sequences and fragments before iterating through the BiRank algorithm. For our purposes, we consider **p** to be the fragments vector and **u** to be the sequences vector. The fragments’ query vector values are calculated using Equation 2:

(2) $$p_{x}^{0}=\frac{I_{x}}{G_{x}^{r2}}$$

For fragment *x*, we assign the query value as its intensity divided by its GAGfinder G-score. The tuning parameter *r2* controls the effect the G-score has on the overall score. The sequences’ query vector values are calculated using Equation 3:

(3) $$u_{y}^{0}={\left(\prod_{m}score_{m}\right)}^{r3}$$

For sequence *y*, we assign the query value as the product of the residue likelihood scores for each monosaccharide residue in the sequence. The residue likelihood is calculated using Equation 4:

(4) $$\begin{array}{l} score_{m}=1.0-0.6*N_{m}-0.3*S_{m}\\ N_{m}=\left\{ \begin{array}{cc} 1 & \text{if}\;\text{amine}\;\text{is}\;\text{unoccupied}\\ 0 & \text{otherwise} \end{array}\right.\\ S_{m}=\left\{ \begin{array}{cc} 1 & \text{if}\;3-O-\text{sulfation}\;\text{without}\;6-O-\text{sulfation}\\ 0 & \text{otherwise} \end{array}\right. \end{array}$$

Each residue has a maximum likelihood value of 1.0. If the residue is an amino sugar that has a free amine group, the value is decreased by 0.6. If the residue is an HS GlcN residue that is 3-*O*-sulfated and not also 6-*O*-sulfated, the value is decreased by 0.3. These deductions account for the rarity of free amines and 3-*O*-sulfation without 6-*O*-sulfation in nature. The tuning parameter *r3* controls how much a sequence with rare modification patterns is punished prior to running the BiRank algorithm. The hyper-parameters α and β control how much of each iteration’s ranking score is due to the query vectors for the fragments and sequences, respectively. A larger value for either hyper-parameter weights the iterating results of BiRank more than the query vector. Once the BiRank algorithm iterates to convergence, GAGrank outputs the ranking of sequences with their ranking score into a tab-delimited file. A larger score represents a higher ranking.

### Data Acquisition and Preprocessing

**Fig. 4:**
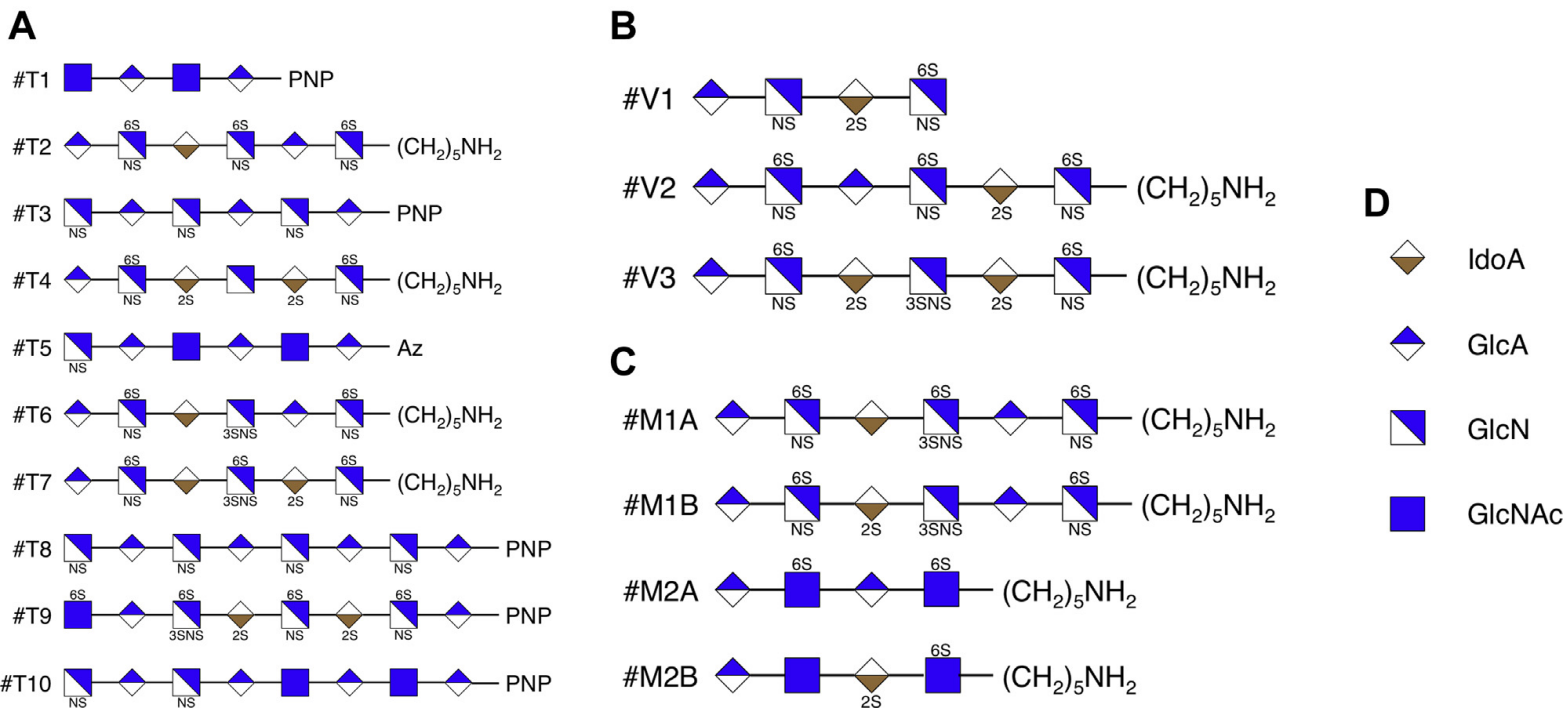
**Structures analyzed in this study.***A*, the ten training saccharides. *B*, the three validation saccharides. *C*, the two isomeric mixtures. *D*, key for the symbols in the figure. Each analyzed structure was dissociated *via* negative electron transfer dissociation, except training compounds #T6 and #T7, which were dissociated *via* electron detachment dissociation. The precursor charge states for these compounds range from −2 to −6. The compounds were selected to represent diversity in chain length, modification amounts and patterns, and charge state for glycosaminoglycan oligosaccharides.

We selected 13 pure synthetic GAG standards on which to train and validate GAGrank and two isomeric mixtures of pure synthetic GAG standards to show GAGrank’s performance on mixtures, shown in Figure 4. These samples were selected for their varying lengths, modification amounts and patterns, disaccharide order, and precursor charge. Ten pure synthetic standards were selected as training data, and three pure standards were selected as validation data. Training compounds T1, T3, T5, T8, T9, and T10 were acquired through a publicly available set of HS standard saccharides funded by the NIH and maintained by the Zaia laboratory (http://www.bumc.bu.edu/zaia/gag-synthetic-saccharides-available/). The remaining training compounds, all of the validation compounds, and the compounds mixed in the isomeric mixtures were synthesized as described (42, 43, 44, 45). Each of the two mixtures was tested in ratios of 100:0, 90:10, 70:30, 50:50, 30:70, 10:90, and 0:100.

Each sample was subjected to either EDD or NETD using a Bruker solariX 12T FTMS instrument. The spectra were converted to centroided mzML using the compassXport command line utility 3.0.13 (Bruker Daltonics, Inc). Elemental compositions of tandem mass spectral peaks corresponding to GAG fragments were determined using a modified version of GAGfinder that requires an isotopic distribution to have peaks A and A + 1 to have an intensity above the noise threshold, with error tolerance of 5 parts per million (ppm) or better and considered sulfate losses determined by the floor of two times the free proton index. The mass spectrometry glycomics data have been uploaded to the GlycoPost repository with the dataset identifier [GPST000014].

### Parameter Optimization

In order to determine optimal values for the above parameters, we employed the simulated annealing (SA) probabilistic optimization procedure. SA is named after the process of annealing in metallurgy whereby material is heated to the point where its geometric structure breaks down and it can be shaped, followed by a slow cooling to reestablish the geometric structure. SA works by randomly moving from one solution to a neighboring solution until a very good, although not necessarily perfect, solution is found. During the course of the SA algorithm, if the new solution has a better fitness than the current solution, the new solution will always be selected; however, if the new solution has a worse fitness than the current solution, the new solution may be selected based on a probabilistic criterion. Equation 5 shows the formula for calculating the probability of moving to a worse solution:

(5) $$P\left(move\right)=e^{\frac{f_{new}-f_{current}}{T}}$$

Here, *f*_*new*_ and *f*_*current*_ represent the fitness scores for the new solution and the current solution, respectively. *T* represents a “temperature” parameter that is analogous to the cooling process in annealing, described above, and “cools” from a value of 1 to 0. For cases where the new solution’s fitness is worse than the current solution’s, when *T* is close to 1, the algorithm will probably still move to that solution, whereas when *T* is close to 0, the algorithm will probably stay at the current solution. Therefore, at the beginning of the algorithm, it is more likely to move to a worse solution than toward the end. This helps combat the problem of local maxima. In addition, each time a new solution produces a fitness value that is higher than any previous fitness value, that solution is stored and may be returned to later if no other solution produces a fitness value as high.

In our implementation, we employed SA to find a very good solution for GAGrank on our ten training oligosaccharides. We optimized the five aforementioned parameters as well as the number of fragments returned by GAGfinder. To identify a new solution we randomly selected one of the six parameters and randomly changed its value. For α and β, the value could be any number between 0 and 1. For *r1*, *r2*, and *r3*, the value could be any number between 0 and 10. For the number of fragments used, the value could be any integer between 5 and 100, or all of the found fragments. We rounded the value for α and β to the hundredth decimal point, and we rounded the value for *r1*, *r2*, and *r3* to the 10th decimal point. We reduced *T* by multiplying it by 0.9. In order to slow the SA process and more completely explore the search space, we remained at each value of *T* for 100 iterations. We calculated the fitness of each solution as the average percent of incorrect sequences with a worse BiRank score than the correct sequence. For example, if a composition has ten possible sequences, the solution’s fitness is equal to 1.0 (9/9) if the correct sequence has the highest BiRank score, 0.0 (0/9) if the correct sequence has the lowest BiRank score, and 0.778 (7/9) if the correct sequence has the third-highest BiRank score.

## Results

### Optimal Parameters

Parameter optimization *via* SA found multiple combinations of parameters that resulted in optimal performance on the training data, which are found in supplemental Table S1 and summarized in Table 1. These combinations all returned a fitness value across the ten training compounds of 0.9997, meaning that, on average, GAGrank returned a better ranking score for the correct sequence than 99.97% of incorrect sequences. Each parameter combination follows similar patterns: large values for *r1* and *r2*, small values for *r3*, large values for α and β, and between 61 and 97 GAGfinder fragments used. The large values for *r1* can be interpreted as evidence that internal double glycosidic bond fragments are far less important for GAG sequencing than terminal fragments. The large values for *r2* can be interpreted as evidence that the fragments’ goodness-of-fit G-scores from GAGfinder are more important factors in GAG sequencing than their intensities. The small values for *r3* can be interpreted as evidence that rare modifications do not need to be punished severely for sequences without rare modifications to perform well. The large values for α and β can be interpreted as evidence that the initial ranking scores for the fragments and sequences are much less important to the optimal performance than the placement and widths of the edges in the graph structure. Finally, the range for the number of fragments to input into GAGrank mostly relates to the number of fragments initially found by GAGfinder; for some saccharides, GAGfinder found fewer than 60 fragments in the spectrum, whereas for others, GAGfinder found well over 100. The range of 60 to 70 suffices to get positional detail for modifications without introducing false positives. In the GAGrank code, we set the default values to the medians in Table 1. Exact values for each combination of parameters are in supplemental Table S1.

**Table 1:** Summary statistics for each GAGrank parameter that resulted in the best performance on the training compounds

Statistic	*r1*	*r2*	*r3*	α	β	# Fragments
Minimum	0.5	0.9	0.1	0.77	0.76	61
Maximum	9.8	9.6	1.7	0.99	1.00	97
Mean	5.5	5.2	0.6	0.93	0.92	70
Median	5.4	5.1	0.4	0.98	0.94	68
Mode	9.3	4.9	0.1	0.98	0.94	64

Table 2 shows the overall ranking and percent of incorrect sequences outscored for each of the training compounds in GAGrank, and supplemental Tables S3–S12 show the GAGrank outputs for each. For eight of the ten training compounds, GAGrank returned the correct sequence with the best ranking score of all of the possible sequences. In training compound #T6, GAGrank returned the correct sequence tied with two other sequences for the second-best ranking score of all of the possible sequences. This is likely due to the effect that training compound #6’s rare 3-*O*-sulfation without 6-*O*-sulfation has on the prior sequence rankings; indeed, supplemental Table S8 shows that the top four sequences all differ only by the presence (or absence) and location of the 3-*O*-sulfation in the sequence. In training compound #T9, GAGrank returned the correct sequence tied with 25 other sequences for the best-ranking score of all of the possible sequences, as seen in supplemental Table S11. This is likely due to the large number of possible sequences for that particular composition, combined with the relative dearth of fragments found by GAGfinder.

**Table 2:** GAGrank performance for the training compounds using any of the optimal parameter combinations

Training compound	Ranking	% Incorrect outscored
#T1	#1 of 2	100
#T2	#1 of 1848	100
#T3	#1 of 440	100
#T4	#1 of 1584	100
#T5	#1 of 60	100
#T6	#2-#4 of 1848	99.8
#T7	#1 of 990	100
#T8	#1 of 3640	100
#T9	#1-#26 of 23,298	99.9
#T10	#1 of 1092	100

### Parameter Validation

Table 3 shows the ranking score, overall ranking, and percentile of each of the validation compounds in GAGrank, and supplemental Tables S13–S15 show the GAGrank outputs for each. Similar to the results for the training compounds, GAGrank returned the correct sequence with the best ranking score of all of the possible sequences, while GAGrank returned the correct sequence tied with one other sequence for the third-best ranking score of all of the possible sequences for the compound with a single 3-*O*-sulfation without a 6-*O*-sulfation. As can be seen in supplemental Table S15, for validation compound #V3, the two sequences with a better ranking score than the actual sequence did not have any rare modifications, whereas the actual sequence and the incorrect sequence with which it tied both have one residue with 3-*O*-sulfation without 6-*O*-sulfation. Unlike the results for training compound #T6, one of the two sequences with a better ranking score than validation compound #V3 did not have the correct modification numbers at each residue in the sequence; the sequence that had the second-best ranking score placed a sulfate at the 2-*O* position of the nonreducing end GlcA rather than at the 6-*O* position of the neighboring GlcN.

**Table 3:** GAGrank performance for the validation compounds using any of the optimal parameter combinations

Validation compound	Ranking	% Incorrect outscored
#V1	#1 of 140	100
#V2	#1 of 1584	100
#V3	#3-#4 of 990	99.7

### GAGrank and GAG Mixtures

Figure 4*C* shows the structures of two pairs of saccharide isomers used to show the ability of GAGrank to analyze mixtures. Table 4 shows the rankings for each compound in each of the two mixtures at each of the ratios and supplemental Tables S16–S29 show the GAGrank outputs for each. As in the training compounds and validation compounds, one of the sequences, mixture compound #M1B, has a rare modification, 3-*O*-sulfation without 6-*O*-sulfation. Furthermore, this sequence never has the best ranking score at any mixture ratio, just as in the similar cases in the training compounds and validation compounds. The sequences corresponding to the remaining three compounds have the highest-ranking score when they comprise at least 70% of the isomeric mixture of which they are a part. Furthermore, each of the compounds used in the mixtures performs as well as it does when it is pure as long as it is 70% or more of the isomeric mixture.

**Table 4:** GAGrank performance for the mixture compounds using any of the optimal parameter combinations

Mixture and ratio	Compound A rank	Compound A % incorrect outscored	Compound B rank	Compound B % incorrect outscored
#M1 100:0	#1 of 1584	100	--	--
#M1 90:10	#1 of 1584	100	#10-#11 of 1584	99.4
#M1 70:30	#1 of 1584	100	#8-#9 of 1584	99.6
#M1 50:50	#22 of 1584	98.7	#2-#3 of 1584	99.9
#M1 30:70	#64 of 1584	96.0	#2-#4 of 1584	99.8
#M1 10:90	#109 of 1584	93.2	#2-#4 of 1584	99.8
#M1 0:100	--	--	#2-#4 of 1584	99.8
#M2 100:0	#1 of 30	100	--	--
#M2 90:10	#1 of 30	100	#6 of 30	85.7
#M2 70:30	#1 of 30	100	#5 of 30	89.3
#M2 50:50	#2 of 30	100	#1 of 30	100
#M2 30:70	#3 of 30	96.4	#1 of 30	100
#M2 10:90	#4 of 30	92.9	#1 of 30	100
#M2 0:100	--	--	#1 of 30	100

### Runtime Analysis

Information about the runtime of GAGrank on each of the compounds and mixtures is available in supplemental Table S2. With the exception of training compound #T9, whose composition has 23,298 different possible sequences, GAGrank ran to completion in under 17 s for each compound, with many running to completion in under 10 s. There is a strong relationship between the number of possible sequences for a compound’s composition and the runtime. GAGrank was tested on a 2011 MacBook Pro that has a 2.4 GHz Intel Core i5 processor with 4 GB RAM. GAGrank should run even faster on a more modern machine with greater computational resources.

## Discussion

Here, we have presented our work on bipartite network representations and analyses for the relationship between GAG sequences and MS^2^ fragment ions, GAGrank. GAGrank is an algorithm that ranks nodes using the bipartite network’s structure and prior information about the sequences and fragments, giving each node an importance score that is derived based on how that fragment fits into the sequence. GAGrank is currently available in command line form. We plan to merge it and our previous work, GAGfinder (18), into a GAG sequencing pipeline in the near future. The command line interface is easy to use, with only a few arguments required for operation.

To our knowledge, this is the first time this approach has been used for the problem of GAG sequencing, and it has certain inherent advantages. One such advantage is that the concept of a relationship between sequences and fragments is intuitive and easy to visualize. Another advantage is that bipartite networks have been exhaustively studied in other fields, meaning that methods for analyzing them have already been developed. GAGrank, at its most basic level, is simply an implementation of one of these methods, BiRank (39). Furthermore, enumerating every sequence that is possible for a given GAG composition allows for ranking sequences by their importance in the network, which is analogous to their likelihood. It is important to note that GAGrank is not a simple fragment counting method but relies on the nimble techniques associated with network analyses, and product fragments score differently depending on the sequence to which they are connected.

We used three separate sets of GAG compounds for training and validation. We optimized GAGrank’s parameters using the ten compounds in our training set and found numerous sets of parameters that returned a near-optimal solution. Using these parameters, we tested GAGrank’s performance on the three compounds in our validation set, and GAGrank returned a similarly near-optimal solution for these compounds. We also tested GAGrank’s ability to sequence GAG mixtures on two separate isomeric mixtures that differed only in one positional sulfation. On these mixtures, GAGrank performed well, ranking the sequence that made up more of the mixture highly while ranking the sequence that made up less of the mixture lower. An intuitive way to view GAGrank’s performance on mixtures is that, the higher the percentage of the mixture a particular sequence is, the higher that sequence ranks. Although GAGrank’s performance on mixtures shows that this method has potential for characterizing mixture constituents, there is currently no means by which users can determine that their sample is a mixture.

For the cases in the training set, validation set, and mixture set where the actual sequence did not rank highest of all the possible sequences, each compound had a rare modification (3-*O*-sulfation without 6-*O*-sulfation on a glucosamine residue) that was penalized in the sequences’ query vector. A simple solution to this problem would be to not punish sequences with rare modifications, but we hypothesize that this would penalize the final performance of sequences that are much more common in nature. In the course of parameter optimization, an α equal to 1.00 was tested numerous times but never returned the best solution. This case (α = 1.00) means that the sequence ranking is derived entirely from the graph structure, without any input from the query vectors. Without a near-full complement of fragments in the spectrum, there will be many sequences that have the exact same edges, and without prior information, GAGrank cannot distinguish them. We believe that the benefit of teasing out the exact correct sequence when it has no rare modifications outweighs the slightly worse performance for those sequences that do have a rare modification. This argues for the use of enrichment steps to increase the concentration of rare modifications in the sample.

There are a couple of unique aspects to GAGrank that may fundamentally alter how GAG sequence analysis is performed, both of which are mostly about user preference. The first is that it requires a peak list from GAGfinder that contains correctly fit elemental compositions, and will not work on peak lists exported from the vendor MS^2^ software generated using averagine approximations. Although this adds an extra step into the pipeline that other programs may not have, it uses the most appropriate means of assigning monoisotopic peaks and elemental compositions. We have demonstrated the efficacy and speed of GAGfinder in that project’s article (18). Another is that GAGrank was not developed to work on metal cationized compounds. Wolff and colleagues were the first group to show how metal cationization reduces sulfate loss for EDD-dissociated HS compounds (46), and this approach succeeds in this endeavor. However, including saccharide ions that have been cationized can severely increase the search space, making the sequencing problem intractable. Furthermore, none of the samples in this article was cationized, and GAGrank performed well even with the higher amounts of sulfate loss.

Of course, GAGrank was tested on pure synthetic saccharides, but biological data are typically noisy and not pure. A typical experiment that generates biological GAG data uses liquid chromatography-tandem mass spectrometry (LC-MS^2^). In LC-MS^2^, samples can be separated in the LC column based on their different physiochemical properties, including charge, size, shape, and hydrophilicities, and an online mass spectrometer generates MS^2^ spectra as samples elute off of the column. This results in a large number of spectra that contain mixtures of GAG structures. We demonstrated GAGrank's performance on mixtures of pure chemicals and showed that there is potential there, but GAGrank is not yet ready to handle such large amounts of high-throughput data and it does not perform as well on mixtures as it does on pure samples. For biological GAG samples, the use of on-line liquid chromatography separations will provide a degree of saccharide purification that is compatible with ExD tandem mass spectrometry (21). The use of ion mobility separations has also been demonstrated with GAG saccharides (22, 47). We envision a combination of on-line LC with ion mobility separations as a means to provide separation of GAG saccharide positional isomers prior to the tandem MS step.

In conclusion, GAGrank demonstrates excellent performance in the difficult task of GAG sequencing. It ranks sequences accurately based on the complement of fragments found *via* GAGfinder and will be a valuable resource for GAG researchers who need fine structure detail for their samples.

## Data Availability

The mass spectrometry glycomics data have been uploaded to the GlycoPost repository (https://glycopost.glycosmos.org/) with the dataset identifier [GPST000014].

## Supplemental data

This article contains supplemental data.

## Conflicts of interest

The authors declare no competing interests.
